# Exercise frequency during the COVID-19 pandemic: A longitudinal probability survey of the US population

**DOI:** 10.1016/j.pmedr.2021.101680

**Published:** 2021-12-27

**Authors:** Indy Wijngaards, Borja del Pozo Cruz, Klaus Gebel, Ding Ding

**Affiliations:** a Tilburg School of Social and Behavioral Sciences, Tilburg University, Tilburg, Netherlands; b Department of Sport Science and Clinical Biomechanics, University of Southern Denmark, Odense, Denmark; c Australian Centre for Public and Population Health, School of Public Health, Faculty of Health, University of Technology Sydney, Ultimo, New South Wales, Australia; d Prevention Research Collaboration, Charles Perkins Centre, Sydney School of Public Health, University of Sydney, Camperdown, New South Wales, Australia

**Keywords:** COVID-19, Exercise, Physical activity, Population representative survey, Public health policy

## Abstract

Regular physical activity is important for general health and reduces the risk for COVID-19 infections and for severe outcomes among infected people. However, measures to mitigate COVID-19 likely decrease population physical activity. This study aimed to examine 1) changes in exercise frequency in a representative sample of US adults during the pandemic (04/01/2020–07/21/2021), and 2) how sociodemographic characteristics, pre-COVID health-related behaviors and outcomes, and state-level stringency of COVID-19 containment measures predict exercise frequency. Self-reported exercise frequency and its individual-level predictors were determined based on 151,155 observations from 6,540 adult participants (aged ≥ 18 years) in all US states from the Understanding America Study. State-level stringency of COVID-19 control measures was examined from the Oxford COVID-19 Government Response Tracker. Exercise frequency varied significantly over 28 survey waves across 475 days of follow-up (*F*_1,473_ = 185.5, *p* < 0.001, η^2^ = 0.28, 95% CI = 0.23-1.00), where exercise frequency decreased between April 2020 and January 2021, and then increased from January 2021 to July 2021. Those who were younger, living alone, non-White, had no college degree, lower household income, low pre-pandemic physical activity levels, obesity, diabetes, kidney disease and hypertension had lower exercise frequency. State-level stringency of COVID-19 control measures was inversely associated with exercise frequency (B = 0.002, SE = 0.001, *p* < 0.01) between April and December 2020 when the overall stringency level was relatively high; but the association was non-significant (B = 0.001, SE = 0.001, *p* > 0.05) between January and July 2021, during which the stringency index sharply declined to a low level. This longitudinal probability survey of the US population revealed significant fluctuations in exercise during COVID-19. Low exercise levels are concerning and deserve public health attention. Health inequalities from physical inactivity are likely to exacerbate because of COVID-19. Physical activity promotion in safe environments is urgently warranted, especially in at-risk population subgroups.

## Highlights

• First longitudinal study based on population representative sample to examine the effects of COVID-19 mitigation measures on exercise.
• Stringent COVID-19 containment measures pose an obstacle for exercise when the overall levels of restrictions were high.
• Health inequalities from physical inactivity might exacerbate due to COVID-19.
• Safely promoting physical activity to at-risk groups is urgently warranted during the COVID-19 pandemic.

## 1 Introduction

COVID-19 poses a major threat to global health (Dong et al., 2020). To control the spread of the virus, governments have been enforcing restrictions such as stay-at-home orders, closures of non-essential businesses, limits on public gatherings, and obligatory face masks (Anderson et al., 2020, Hale et al., 2020). While such restrictions reduce infections (Koo et al., 2020), some likely come with public health costs, such as declining physical activity (Holmes et al., 2020, Caputo and Reichert, 2020, Beck et al., 2021, de Lannoy et al., 2020). For example, two international studies and one Chinese study based on convenience samples and smartphone accelerometers respectively wrist-worn activity trackers reported decreases in daily step counts of 25% to 54% at the start of the pandemic (Tison et al., 2020, Pépin et al., 2020, Ding et al., 2021).

Declining physical activity is a major public health concern. Regular activity is critical to the prevention and management of non-communicable diseases, such as coronary heart disease, type 2 diabetes, and some cancers (Lee et al., 2012, Lear et al., 2017, US Department of Health and Human Services, 2018, World Health Organization, 2020), and has been considered today’s ‘best buy in public health’ (Morris, 1994). Physical activity improves immune function (Nieman and Wentz, 2019), and emerging evidence suggests that regular activity lowers COVID-19 infection risk (Chastin et al., 2021), hospitalization (Hamer et al., 2020) and severe COVID-19 outcomes among those infected (Sallis et al., 2020, Ding et al., 2020). Moreover, physical activity has mental health benefits (Ding et al., 2020, Buecker et al., 2021) which is especially important considering the alarming impact of the pandemic on population mental health and wellbeing (Rajkumar, 2020). A rapid meta-analysis of 21 studies showed that regular physical activity was associated with lower odds of depressive symptoms and anxiety during the COVID-lockdown (Wolf et al., 2020).

Dozens of studies have examined physical activity levels during the COVID-19 pandemic. However, nearly all were cross-sectional and conducted in the first few weeks of the pandemic, when most governments implemented strict measures for the first time, which led to radical changes in lifestyles “overnight”. Therefore, findings from these studies may become less relevant at later stages of the pandemic (e.g., new waves of infections, adaptation effects, ‘behavioral fatigue’, relaxation of COVID-19 protective measures, vaccination). Hence, it remains unclear how activity levels have changed since the start of the COVID-19 pandemic. Furthermore, apart from one cross-sectional study in Germany (Beck et al., 2021), no studies used probability-based sampling, limiting the generalizability of the existing evidence. To the best of our knowledge, our study is the first longitudinal and population representative study on the effects of COVID-19 mitigation measures on leisure-time physical activity (a.k.a., exercise). Finally, although lockdown measures differ dramatically around the world and across different states in the US and over time (Hale et al., 2020), which would likely affect physical activity differently (López-Bueno et al., 2020, Stables, 2020), we are only aware of two cross-sectional studies that have examined the association between the stringency of COVID-19 containment measures and activity levels. One study found a non-significant negative relationship between the stringency of mitigation policies in the different states of Germany and activity levels in people aged 14 and above (Beck et al., 2021). Another study found that children and adolescents in Canadian provinces with the strictest mitigation policies had the largest declines in time spent outdoors and outdoor play (de Lannoy et al., 2020).

Using data from a longitudinal probability survey of the US population, this study addresses two research questions: (1) to investigate trends in leisure-time exercise frequency during the COVID-19 pandemic (between April 1, 2020, and July 21, 2021), (2) to identify predictors of exercise frequency during the COVID-19 pandemic, including sociodemographic characteristics, health-related behaviors and outcomes, and stringency of state-level COVID-19 containment measures.

## 2 Methods

### 2.1 Sampling and procedures

Data were from the Understanding America Study (UAS), a nationally representative panel of 9,063 adults (aged ≥ 18 years) from all US states that began in 2014, conducted by the Center for Economic and Social Research at the University of Southern California (Kapteyn et al., 2020). Participant addresses were from the US Postal Service Delivery Sequence file and a three-stage sampling methodology was used (Kalton et al., 2014). Participants completed the survey on a computer, mobile device, or tablet. Internet-connected tablets were offered to households without internet access.

Participants provided informed consent prior to participation and received a $10 compensation for completing the first COVID-19 survey, the UAS 230, which was administered between March 10 and 31, 2020, followed by repeated surveys for which participants received a compensation of $13 each. For the current research, we used data from 28 follow-up surveys, fielded between April 1–14, 2020 (UAS 235) and June 9 – July 21, 2021 (UAS 348). We did not use the initial survey because of incompatible exercise measures. Detailed information about the UAS can be found in a methods paper (Angrisani et al., 2019), and online (https://UASdata.usc.edu). The UAS was approved with waiver of informed consent by the Institutional Review Board of the University of Southern California (IRB no. 18-00796).

### 2.2 Measures

Exercise frequency was measured by asking: “Out of the past 7 days, what is your best estimate of the number of days that you got exercise?” This question was based on previously validated single-item measures that assess leisure-time physical activity (Scott et al., 2015, Milton et al., 2011, Iwai et al., 2001). For example, a UK study showed that a similar single-item measure agreed strongly with classifying respondents as sufficiently active (κ = 0.63, 95% CI 0.54–0.72) and had good 2- to 5-day test–retest reliability (*r* = 0.72–0.82) (Milton et al., 2011).

Demographic and socioeconomic variables were extracted from the UAS Household Survey (i.e., age group, sex, race, college degree, living with partner, employment status, household income). Pre-pandemic physical activity frequency was extracted from the UAS 185, collected in June 2019. Three questions asked about the frequency of vigorous-, moderate- and light-intensity physical activity. Response categories were 1 (more than once a week), 2 (once a week), 3 (one to three times a month) and 4 (hardly ever or never). The three items have been used in other longitudinal panel surveys such as the Health and Retirement Study (Juster and Suzman, 1995) and the National Longitudinal Survey of Adolescent to Adult Health (ARDA, 2018). In line with previous research (Feng et al., 2016, Germain et al., 2016), we dichotomized each variable as active (more than once a week) versus inactive (once a week or less). We recoded missing values on this question into “no data” because list-wise deletion of respondents would otherwise result in a sample size reduction of 1628 (24.9%). In the UAS 185, participants were also asked to report whether a health professional had diagnosed them with diabetes, cancer, obesity, heart disease, high blood pressure, asthma, chronic lung disease, kidney disease, autoimmune disorder or mental health conditions.

Data on the stringency of COVID-19 containment measures in US states was extracted from the Oxford COVID-19 Government Response Tracker (OxCGRT) (Hale et al., 2020), which provides a systematic tracking of responses to COVID-19 across states. We used the OxCGRT’s Standardized Stringency Index, a dynamic, continuous index composed of eight ordinal variables regarding COVID-19 restrictions: school and workplace closures, restrictions of public events, gathering sizes, public transport, and domestic/international travel, and ‘stay at home’ orders. This time-varying measure ranged from 0 (no measures) to 100 (total lockdown) and was linked with the UAS data based on participants’ date of survey completion and state of residence.

### 2.3 Statistical analysis

**Fig. 1:**
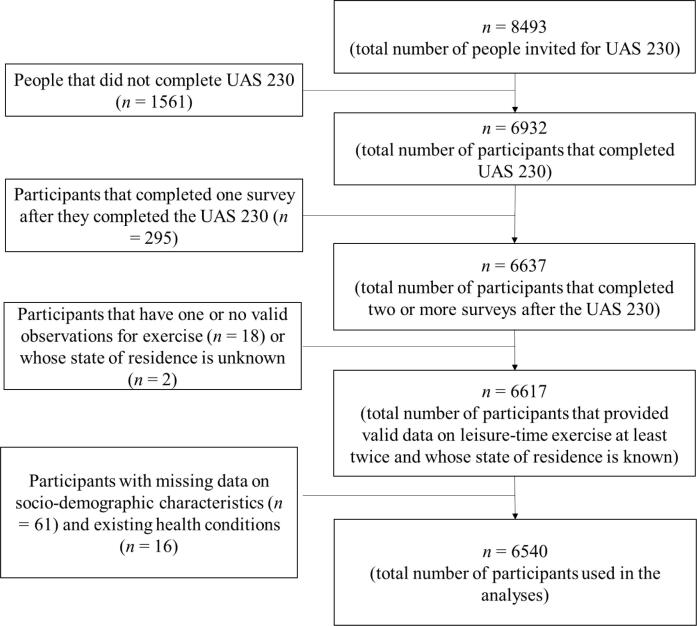
Participant flowchart *Notes.* UAS = Understanding America Study; UAS 230 = first COVID-19 survey in UAS administered during March 10-31, 2020.

As shown in Fig. 1, we excluded participants who did not provide valid data on exercise at least twice since the UAS 230, those whose state of residence was unknown, and those who did not provide complete data on sociodemographic variables or health outcomes. This selection procedure resulted in a final sample of 151,155 observations from 6,540 participants.

We conducted two analyses to answer the two research questions. For the first analysis, we examined changes in the average number of days of exercise per week from April 1, 2020, to July 21, 2021. Exercise frequency trends were visualized as mean and 95% CI by survey date using a locally weighted scatterplot smoothing curve. Analysis of variance (ANOVA) was used to determine whether exercise levels differed significantly over time. Effect sizes were computed based on partial eta squared (η^2^*_p_*). A regression discontinuity analysis was used to quantify the significance of fluctuations in the trend line.

For the second analysis, we fitted a set of three nested random effects models to delineate the influence from different blocks of predictors while accounting for the hierarchical nature of the data (observations nested within individuals). Specifically, we estimated effects of demographic and socio-economic characteristics in Model 1, while sequentially adding health-related behaviors and outcomes in Model 2, and the COVID-19 OxCGRT stringency index in Model 3. We conducted two sensitivity analyses when handling the pre-COVID-19 physical activity question. First, we re-fitted the three models using a different cut-off point: ‘once a week or more’ vs ‘less than once a week’, in order to address the lack of an evidence-based cut-off point for this question. Second, instead of using a missing indicator approach, we used complete case analysis in the sample with complete data on the pre-COVID-19 physical activity question (115,627 observations from 4,912 participants). We ran an additional sensitivity analysis when examining the association between stringency of COVID-19 restrictions and exercise frequency by conducting sub-analysis for the association during the period when the stringency of restrictions was high and the period when most restrictions started to be lifted.

Participant weights provided by the UAS were used to adjust for the complex survey design, non-response rate, unequal selection probabilities and non-random attrition across waves (Angrisani et al., 2019). The sample weights were computed based on the general two-step UAS Weighting Procedure. First, base weights were computed, which correct for unequal probabilities of sampling UAS panel members. Second, we computed the final post-stratification weights, which ensure that the sample aligns with the reference population regarding gender (male, female), race and ethnicity (White, Black, Other, Hispanic, Native American), age (18–39, 40–49, 50–59, 60+ ), education (high school or less, some college, Bachelor or more), Census regions (Northeast, Midwest, South, West, excluding Los Angeles County). The benchmark distributions were based on the six most recent available Current Population Survey (CPS) Basic Monthly Surveys with respect to the survey's completion dates. We present unstandardized regression coefficients and standard errors. All models were adjusted for survey wave and state of residence and alpha was set at 0.05. All analyses were conducted in R version 3.6.1 (R Core Team, 2018) (codes provided in the Supplementary materials). The study complies with the Strengthening the Reporting of Observational Studies in Epidemiology (STROBE) guidelines for cohort studies (von Elm et al., 2014).

## 3 Results

### 3.1 Sample description

**Fig. 2:**
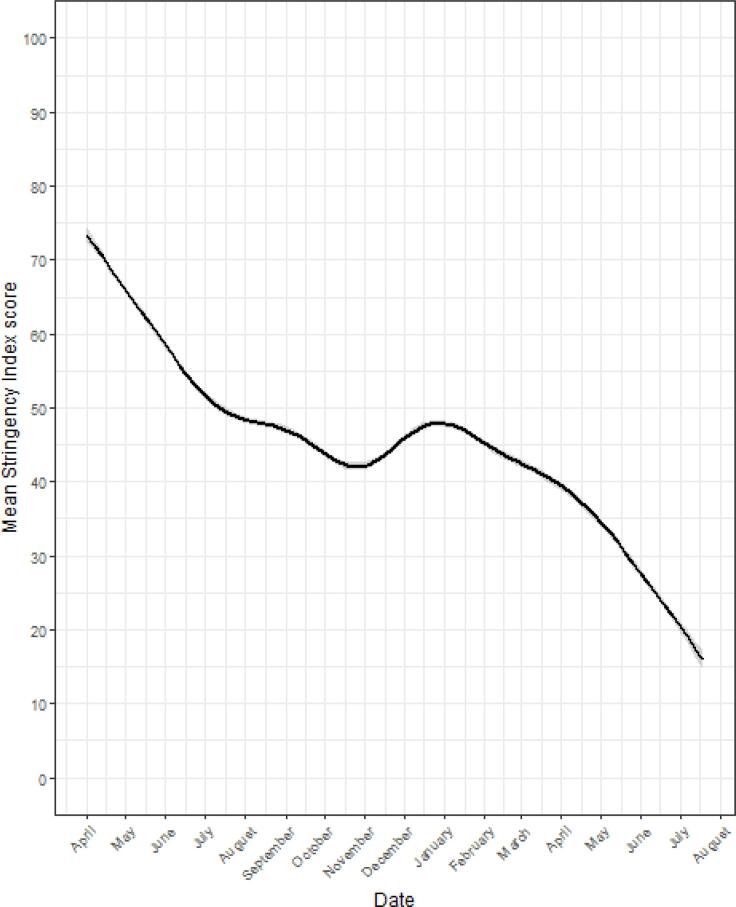
Stringency index score in 50 US states between 01/04/2020 and 07/21/2021.

As shown in Table 1, 58.7% of the participants were female, 59.8% were aged 45 years and above, 83.0% were White, 55.6% had a college degree, 54.2% lived with a partner, 59.6% were employed and 66.3% had a household income of $40,000 or more. Participants that completed the UAS 185 in 2019 were reasonably active before the pandemic started, with 66.9%, 53.0% and 33.1% engaging in light, moderate and vigorous physical activity more than weekly (24.8% did not complete the UAS 185). High blood pressure (31.4%), obesity (17.9%), and diabetes (12.0%) were the most prevalent health conditions, while autoimmune disorder (6.1%), chronic lung disease (4.1%), and kidney disease (2.6%) were the least common (Table 1). The stringency of COVID-19 measures varied substantially over time (*F*_1,473_ = 1442, *p* < 0.001, η^2^*_p_* = 0.75, 95% CI =0.75-1.00). As illustrated in Fig. 2, measure stringency levels steadily declined from April 2020 to October-November 2020, increased slightly from November 2020 to January 2021 and declined sharply from January to July 2021.

**Table 1:** Sample characteristics (*N* = 6,540).

Variable	*n*	%	Mean #days leisure-time exercise per week	SD #days Leisure-time exercise per week	*P*
**Demographic characteristics**					
Age group (years)					**<0.001**
18–24	318	4.9	2.69	2.24	
25–34	991	15.2	2.84	2.29	
35–44	1321	20.2	3.16	2.28	
45–54	1164	17.8	3.21	2.27	
55–69	1902	29.1	3.78	2.31	
≥70	844	12.9	4.20	2.33	
Sex					**<0.001**
Male	2704	41.3	3.74	2.31	
Female	3836	58.7	3.27	2.34	
Race					**<0.001**
White	5425	83.0	3.58	2.33	
Non-White	1115	17.0	2.87	2.30	
College degree					**<0.001**
Yes	3639	55.6	3.65	2.28	
No	2901	44.4	3.22	2.39	
Living with partner					**<0.001**
Yes	3546	54.2	3.67	2.31	
No	2994	45.8	3.20	2.35	
Employment status					**<0.001**
Employed	3897	59.6	3.33	2.29	
Unemployed	1264	19.3	3.01	2.35	
Retired	1379	21.1	4.17	2.29	
Annual household income					**<0.001**
≤$15,000	750	11.5	2.81	2.41	
$15,001-$39,999	1452	22.2	3.21	2.37	
$40,000-$99,999	2638	40.3	3.55	2.32	
≥$100,000	1700	26.0	3.81	2.25	

**Pre-COVID-19 PA frequency**					
Light-intensity					**<0.001**
More than once a week	3077	47.0	3.89	2.28	
Once a week or less	1838	28.1	2.65	2.23	
No data	1625	24.8	3.58	2.33	
Moderate-intensity					**<0.001**
More than once a week	2605	39.8	4.22	2.19	
Once a week or less	2310	35.3	2.51	2.17	
No data	1625	24.8	3.58	2.33	
Vigorous-intensity					**<0.001**
More than once a week	1625	24.9	4.47	2.15	
Once a week or less	3289	50.3	2.91	2.26	
No data	1625	24.8	3.58	2.33	

**Self-reported health conditions**					
Diabetes					**<0.001**
Yes	783	12	3.53	2.34	
No	5757	88	3.04	2.32	
Cancer					**<0.001**
Yes	457	7	3.73	2.37	
No	6083	93	3.44	2.34	
Obesity					**<0.001**
Yes	1168	17.9	2.69	2.23	
No	5372	82.1	3.64	2.33	
Heart disease					**<0.001**
Yes	426	6.5	3.64	2.41	
No	6114	93.5	3.45	2.34	
High blood pressure					**<0.001**
Yes	2053	31.4	3.39	2.34	
No	4488	68.6	3.50	2.34	
Asthma					**<0.001**
Yes	760	11.6	3.26	2.37	
No	5780	88.4	3.49	2.34	
Chronic lung disease					**<0.001**
Yes	266	4.1	2.97	3.38	
No	6274	95.9	3.49	2.34	
Kidney disease					**<0.001**
Yes	171	2.6	3.02	2.45	
No	6369	97.4	3.48	2.34	
Autoimmune disorder					**<0.001**
Yes	398	6.1	3.27	2.34	
No	6142	93.9	3.48	2.34	
Mental health condition					**<0.001**
Yes	740	11.3	3.10	2.33	
No	5800	88.7	3.51	2.34	

*Notes.* PA = physical activity; COVID-19 = Coronavirus disease 2019; SD = standard deviation; *P*-value was calculated based on ANOVA test for difference in number of days of leisure-time exercise per week across subgroups.

### 3.2 Exercise frequency trends

**Fig. 3:**
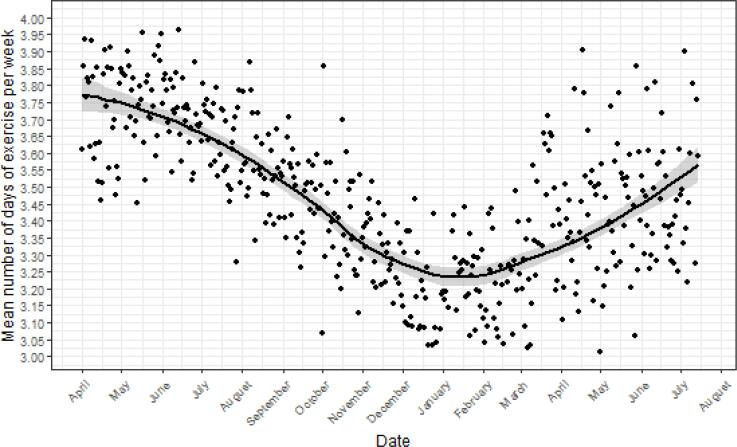
Leisure-time exercise in 6,540 US adults between 01/04/2020 and 07/21/2021*Outliers are due to the relatively low number of observations on specific dates.

The average number of days of exercise per week was 3.47 (SD = 2.34) during the nearly 16 months of follow-up time. There was significant variation in weekly exercise levels over the 28 survey waves (*F*_1,26_ = 42.18, *p* < 0.001, η^2^*_p_* = 0.62, 95% CI = 0.41-1.00) across 475 survey days (*F*_1,473_ = 185.5, *p* < 0.001, η^2^*_p_* = 0.28, 95% CI = 0.23-1.00), with the trend lines, as visualized in Fig. 3, following a U-shape. The average number of days of exercise per week decreased from 3.72 (SD = 2.36) in April 2020, to 3.09 (SD = 2.38) in December 2020 to January 2021, and, subsequently, increased to 3.46 (SD = 2.34) in June-July 2021. The largest drop in exercise levels took place between October 2020 and January 2021. A regression discontinuity analysis on the significance of the slope before and after January 1, 2021, showed that the slopes varied significantly (B = 0.004, SE = 0.000, *p* < 0.001).

### 3.3 Predictors of exercise

We investigated the associations of time-invariant socio-demographic and health behaviors / outcomes and the time-variant state-level Stringency Index with exercise frequency. Model 1 showed that females, and those who were younger than 55, employed, non-White, not living with a partner, without a college degree, or with lower income levels exercised less frequently (Table 2). When including health-related behaviors and outcomes in Model 2, frequent participation in physical activity of any intensity prior to the pandemic was associated with higher frequency of exercise during the pandemic; obesity, diabetes, kidney disease, and high blood pressure were inversely associated with exercise frequency. Meanwhile, living with a partner and education became non-significant. In Model 3, all predictors from Model 2 remained significant and of similar magnitude. Stringency of COVID-19 containment measures was not significantly associated with the frequency of exercise. However, when we re-fitted Model 3 for the two periods of different levels of restrictions, we found that in the period between April and December 2020, the COVID-19 OxCGRT stringency index had a significant inverse association with exercise frequency (B = 0.002, SE = 0.001, *p* < 0.01). During the period between January and July 2021, when the stringency of COVID-19 restrictions quickly declined to a very low level, the COVID-19 OxCGRT stringency index had a nonsignificant association with exercise frequency (B = 0.001, SE = 0.001, p = ns). This finding, combined with the steep and steady downward trend line starting on January 1, 2021, as displayed in Fig. 2, suggests that measure stringency is only an important predictor of exercise frequency if the average level of stringency is relatively high. Finally, as shown in Supplementary Tables 1 and 2, our findings did not change substantially in sensitivity analyses where an alternative cut-off point for the pre-COVID-19 physical activity measure was used and when we limited our analysis to the participants with no missing data on the pre-COVID-19 physical activity measure.

**Table 2:** Unstandardized regression coefficients with standard error (in brackets) from random effects regression models on exercise frequency predictors (n = 6540).

	Model 1	Model 2	Model 3
**Sociodemographic characteristics**			
Age group (years)			
18–24	Reference	Reference	Reference
25–34	−0.089 (0.070)	−0.035 (0.068)	−0.035 (0.068)
35–44	0.089 (0.078)	0.179 (0.074) *	0.179 (0.074) *
45–55	0.225 (0.082) ***	0.389 (0.079) ***	0.389 (0.079) ***
55–69	0.367 (0.083) ***	0.582 (0.081) ***	0.582 (0.081) ***
≥70	0.602 (0.093) ***	0.847 (0.091) ***	0.847 (0.092) ***
Sex			
Male	Reference	Reference	Reference
Female	−0.318 (0.046) ***	−0.213 (0.043) ***	−0.213 (0.043) ***
Race			
White	Reference	Reference	Reference
Non-White	−0.365 (0.064) ***	−0.276 (0.058) ***	−0.276 (0.058) ***
College degree			
Yes	Reference	Reference	Reference
No	−0.224 (0.044) ***	−0.022 (0.041)	0.022 (0.041)
Living with partner			
Yes	Reference	Reference	Reference
No	−0.123 (0.037) ***	−0.062 (0.035)	0.062 (0.035)
Employment status			
Employed	Reference	Reference	Reference
Retired	0.362 (0.047) ***	0.364 (0.046) ***	0.364 (0.046) ***
Unemployed	0.028 (0.024)	0.053 (0.024) *	0.053 (0.024) *
Household income			
≤$15,000	Reference	Reference	Reference
$15,001–$39,999	0.104 (0.035) ***	0.085 (0.034) *	0.085 (0.034) *
$40,000–$99,999	0.102 (0.038) ***	0.040 (0.038)	0.040 (0.038)
≥$100,000	0.265 (0.046) ***	0.146 (0.045) **	0.146 (0.045) **

**Health-related behaviors and outcomes**			
Light-intensity pre-COVID-19 PA frequency			
More than once a week		Reference	Reference
Once a week or less		−0.454 (0.058) ***	**−0.454 (0.058)**
No data		−0.996 (1.325)	−0.996 (1.324)
Moderate-intensity pre-COVID-19 PA frequency			
More than once a week		Reference	Reference
Once a week or less		0.903 (0.061) ***	0.903 (0.061) ***
No data		−1.801 (1.428)	−1.801 (1.428)
Vigorous-intensity pre-COVID-19 PA frequency			
More than once a week		Reference	Reference
Once a week or less		0.810 (0.059) ***	0.810 (0.059) ***
No data		−1.901 (1.066)	−1.901 (1.066)
Health conditions^a^			
Diabetes		−0.217 (0.070) **	−0.217 (0.070) **
Cancer		0.002 (0.083)	0.002 (0.083)
Obesity		−0.550 (0.058) ***	−0.550 (0.058) ***
Heart disease		**0.176 (0.089)** *	**0.176 (0.089)** *
High blood pressure		−0.157 (0.051) **	−0.157 (0.051) **
Asthma		0.007 (0.065)	0.007 (0.065)
Chronic lung disease		−0.247 (0.109) *	−0.247 (0.109) *
Kidney disease		−0.359 (0.132) **	−0.359 (0.132) **
Autoimmune disorder		−0.056 (0.088)	−0.056 (0.088)
Mental health condition		−0.054 (0.068)	−0.054 (0.068)

**State-level containment measures**			
Oxford COVID-19 Government Response Tracker Stringency Index			−0.000 (0.001)^b^

*Notes.* *p < 0.05; ^**^p < 0.01; ^***^p < 0.001, PA = physical activity.

Model 1 included only sociodemographic variables, Model 2 additionally included health-related behaviors and outcomes; Model 3 additionally included COVID-19 containment measure stringency. Weights provided by the UAS were used to adjust for the complex survey design, non-response rate, unequal selection probabilities and non-random attrition across waves. The Satterthwaite method was applied to the *t*-tests used for significance testing. All models were adjusted for survey wave and state of residence.

a Reference category = does not have the particular health condition.

b Regression coefficient for Stringency Index: April-Dec 2020: (B = 0.002, SE = 0.001, *p* < 0.01); Jan-July 2021: (B = 0.001, SE = 0.001, *p* > 0.05).

## 4 Discussion

Using data from 28 surveys fielded between April 1, 2020, and July 19, 2021, in a large, nationally representative sample of the US population, we found that exercise frequency varied significantly during the pandemic. While weekly exercise frequency decreased between April 2020 and January 2021, it increased between January 2021 and July 2021. Multivariate analyses showed that several sociodemographic characteristics, health behaviors and outcomes were related to exercise frequency during the pandemic. The stringency of COVID-19 containment measures was predictive of exercise frequency only when the levels of restrictions were high. Altogether, these findings suggest that population subgroups, such as younger and non-White adults, females, and those suffering from obesity, diabetes, and high blood pressure, are particularly in need of physical activity promotion programs during the pandemic.

Our study found declines in exercise from an average of 3.72 days/week (SD = 2.36) in April 2020, to 3.09 days/week (SD = 2.38) in January 2021, a 17% reduction. From this period onward, exercise frequency increased steadily to 3.46 days/week (SD = 2.34) in July 2021. This seasonal pattern may also be due to indoor exercise facilities playing a more important role in winter than in summer (Wagner et al., 2019, Tucker and Gilliland, 2007). Considering that most indoor facilities remain closed throughout the pandemic (Bentlage et al., 2020), lower levels of leisure-time physical activity in winter may reflect fewer opportunities for exercising outdoors. Moreover, the ‘re-bounce’ of exercise frequency may also be a result of mass vaccination which started from early 2021 and may have contributed to individuals feeling safer to exercise indoors again.

Our study has identified several population subgroups at risk for insufficient exercise during COVID-19. In terms of sociodemographic characteristics, females, non-White, and lower socioeconomic status were associated with lower weekly exercise frequency. These sociodemographic predictors are consistent with extensive pre-pandemic evidence (Milton et al., 2011, Choi et al., 2017). However, when we additionally adjusted for health-related behavior and outcomes pre-pandemic, the associations involving sex, race, education and marital status were all significantly attenuated (and became non-significant in the case of education and marital status). This suggests that much of the associations observed between demographic characteristics and exercise frequency during COVID-19 could be explained by health-related behaviors and outcomes. Interestingly, our analyses found that those aged 55 years and above and retirees participated in more frequent exercise than their younger and working counterparts. This contradicts findings from most pre-pandemic research, and it is particularly surprising considering that older people are considered more at risk for severe outcomes from COVID-19. Overall, research on changes in physical activity in older versus younger adults during COVID-19 revealed mixed findings. While a longitudinal study from China found more declines in physical activity among older adults (Ding et al., 2021), cross-sectional studies from the UK and Portugal reported that older people were more active than younger adults during the pandemic (Smith et al., 2020, Antunes et al., 2020). A smartphone-tracking study of UK adults showed that younger people were more active before the lockdown and the least active after the lockdown, whereas those aged 65 + years increased their activity levels once the lockdown was relaxed (McCarthy et al., 2021). Our observation could be a result of older adults having more discretionary time during COVID-19. It is important to acknowledge that population subgroups’ exercise behavior in response to COVID-19 is likely to be affected by a range of factors, such as containment policies (e.g., whether outdoor exercise is allowed), cultural norms and social circumstances (e.g., living arrangement of older people, caring responsibility), which may explain different findings observed from different countries.

Our finding that participants who were physically active before the pandemic were more active during the pandemic echoed findings from previous studies. For example, a Canadian study showed that 40.6% of participants who were insufficiently active before the pandemic had a drop in their activity levels during the pandemic, whereas only 22.4% of the previously more active participants became less active (Lesser and Nienhuis, 2020). A German study found that the majority of participants maintained their pre-pandemic activity levels during the outbreak (Mutz and Gerke, 2021). A prospective study from China found that after the initial lockdown, those who were sufficiently active before COVID-19 recovered step counts much faster than their insufficiently active counterparts (Ding et al., 2021). Altogether, these findings raise the concern that health inequalities from physical inactivity are likely to exacerbate as a result of COVID-19. This is further underlined by our finding that participants with lifestyle-related chronic conditions, such as obesity, diabetes, and high blood pressure, were less active during the pandemic than those without these conditions. Indeed, a large body of pre-pandemic research (Chau et al., 2012, Thorp et al., 2011, Prince et al., 2017) and a handful of cross-sectional studies conducted during the pandemic (Giustino et al., 2020, Robinson et al., 2020, Robinson et al., 2021) show that obese people are generally less active. Considering the substantial disease burden from obesity (Bhaskaran et al., 2018) and the elevated risk of severe COVID-19 symptoms and mortality among those who are obese (Klang et al., 2020), it is pivotal to prioritize physical activity promotion among people with the most inactive lifestyles and those who are obese and have chronic conditions.

Finally, this study contributes to the dearth of evidence on the association between COVID-19 containment measures and physical activity levels. Our findings partially supported those from cross-sectional studies in Canada (de Lannoy et al., 2020) and Germany (though not statistically significant) (Beck et al., 2021), and the hypothesis that stringent measures pose a barrier for leisure-time physical activity (Caputo and Reichert, 2020, Beck et al., 2021, de Lannoy et al., 2020, Tison et al., 2020, Pépin et al., 2020). In addition, our findings extended previous research by concluding that the association between COVID-19 restrictions and exercise may depend on the overall levels of stringency of these restrictions. Overall, our finding is supported by qualitative research which found that the closing of gyms, sports clubs, health/rehabilitation centers and swimming pools were the most commonly mentioned reasons for decreases in leisure-time physical activity during COVID-19 (Mutz and Gerke, 2021). Our finding highlights the importance of promoting physical activity during the pandemic, particularly in areas with strict containment policies. For example, in areas where outdoor physical activity is unsafe, it is important to promote exercise in the safety of people’s homes. Previous work showed an unprecedented increase in online interest in exercise at the start of the pandemic, suggesting that the virtual environment could be capitalized to promote home-based physical activity during COVID-19. In areas where outdoor activities are relatively safe, it is important to encourage people to engage in outdoor activities such as walking, running and cycling. In areas with low infection risk, where indoor exercise is currently permitted, it is important that exercise facilities continue to operate under strict public health and safety guidelines to prevent the spread of infectious disease (Amagasa et al., 2020).

### 4.1 Strengths and limitations

This study has several strengths. First, our findings are based on data from a large, nationally representative panel of adults from all 50 states, which enables us to generalize our findings to the US adult population. Second, the longitudinal research design of the UAS study and robust modelling strategy allowed us to better understand effects of individual and environmental factors affecting exercise frequency throughout the pandemic. Third, this is the first longitudinal study to examine the association between stringency of COVID-19 containment measures and exercise frequency, using a dynamic indicator of state-level public health restrictions and policies, which is objective and comparable across states and over time. Different COVID-19 containment policies across the US have provided us with a large variance in the Stringency Index to explore the association between COVID-19 containment measures and exercise frequency.

Some limitations should also be noted. First, as the UAS did not use a comparable measure of pre-pandemic exercise frequency, it was impossible to compare levels before and during the pandemic. Second, like in most large cohort studies, the UAS exercise measure is self-reported and hence subject to social-desirability and recall biases. The measure cannot capture the type or duration of exercise. Theoretically, some study participants could have at least partly compensated for a reduction in the number of days per week with exercise by achieving higher volumes of activity on these days. However, as mentioned in the methods section, single-item physical activity measures similar to the one used in the current study have been found to have good concurrent validity in assessing people’s activity level (Scott et al., 2015, Milton et al., 2011, Iwai et al., 2001). The crudeness of the measure leaves some research questions unanswered. For example, which types and settings (e.g., indoor vs outdoor, group vs solo) of leisure-time physical activity are most strongly affected by the pandemic? Are these effects universal across subpopulations and over time? Third, even though weights were used to adjust for complex survey design, non-response rate, unequal selection probabilities, non-random attrition across waves, missing data forced us to exclude a small proportion of participants from the sample. These exclusion criteria may have affected the generalizability of the sample.

## 5 Conclusions

In conclusion, the present study found a significant variation in exercise frequency in the US during the COVID-19 pandemic, with a decrease in exercise frequency between April 2020 and January 2021 and an increase between January 2021 and July 2021. Females, non-Whites, those not living with a partner and of lower socioeconomic status, those who were insufficiently active, obese, and had high blood pressure and diabetes prior to COVID-19 were particularly at risk for lower exercise frequency. Stringent governmental containment measures were found to be a barrier to exercise frequency only when the overall levels of stringency were high. Surprisingly, older people and retirees were more active during leisure time than their younger and working counterparts. In summary, these findings help public health decision makers and practitioners identify at-risk populations that should be targeted for physical activity promotion during the pandemic.

## Declaration of Competing Interest

The authors declare that they have no known competing financial interests or personal relationships that could have appeared to influence the work reported in this paper.
