# Supplementary material for: Exercise frequency during the COVID-19 pandemic: A longitudinal probability survey of the US population
Source: Prev Med Rep. 2021 Dec 27;25:101680. doi: 10.1016/j.pmedr.2021.101680 (PMC8710431; doi:10.1016/j.pmedr.2021.101680)
Supplement: Supplementary Data 1 [file mmc1.docx]

# **SUPPLEMENTARY FILES**

## **Supplementary Table 1.** Unstandardized regression coefficients with standard error (in brackets) from Sensitivity Analysis 1: Using an alternative cut-off point for pre-COVID-19 physical activity frequency (n=6540)

|  | Model 1 | Model 2 | Model 3 |
| --- | --- | --- | --- |
| **Sociodemographic characteristics** | | | |
| Age group (years) |  |  |  |
| 18-24 | Reference | Reference | Reference |
| 25-34 | -0.089 (0.070) | -0.024 (0.068) | -0.024 (0.068) |
| 35-44 | 0.089 (0.078) | **0.202 (0.074) **** | **0.202 (0.074) **** |
| 45-55 | **0.225 (0.082) ***** | **0.429 (0.080) ***** | **0.429 (0.080) ***** |
| 55-69 | **0.367 (0.083) ***** | **0.646 (0.081) ***** | **0.646 (0.081) ***** |
| ≥70 | **0.602 (0.093) ***** | **0.924 (0.092) ***** | **0.924 (0.092) ***** |
| Sex |  |  |  |
| Male | Reference | Reference | Reference |
| Female | **-0.318 (0.046) ***** | **-0.210 (0.044) ***** | **-0.210 (0.044) ***** |
| Race |  |  |  |
| White | Reference | Reference | Reference |
| Non-White | **-0.365 (0.064) ***** | **-0.316 (0.059) ***** | **-0.316 (0.059) ***** |
| College degree |  |  |  |
| Yes | Reference | Reference | Reference |
| No | **-0.224 (0.044) ***** | -0.068(0.036) | -0.068(0.036) |
| Living with partner |  |  |  |
| Yes | Reference | Reference | Reference |
| No | **-0.123 (0.037) ***** | -0.062 (0.035) | -0.062 (0.035) |
| Employment status |  |  |  |
| Employed | Reference | Reference | Reference |
| Retired | **0.362 (0.047) ***** | **0.390 (0.046) ***** | **0.390 (0.046) ***** |
| Unemployed | 0.028 (0.024) | **0.056 (0.024) *** | **0.056 (0.024) *** |
| Household income |  |  |  |
| ≤$15,000 | Reference | Reference | Reference |
| $15,001–$39,999 | **0.104 (0.035) ***** | **0.094 (0.034) *** | **0.094 (0.034) *** |
| $40,000–$99,999 | **0.102 (0.038) ***** | 0.057 (0.038) | 0.057 (0.038) |
| ≥$100,000 | **0.265 (0.046) ***** | **0.171 (0.046) ***** | **0.171 (0.046) ***** |
| **Health-related behaviors and outcomes** | | | |
| Light-intensity pre-COVID-19 PA frequency | |  |  |
| Once a week or more |  | Reference | Reference |
| Less than once a week |  | **-0.426 (0.073) ***** | **-0.426 (0.073) ***** |
| No data |  | -0.530 (1.347) | -0.530 (1.347) |
| Moderate-intensity pre-COVID-19 PA frequency | |  |  |
| Once a week or more |  | Reference | Reference |
| Less than once a week |  | **-0.829 (0.065) ***** | **-0.829 (0.065) ***** |
| No data |  | 1.525 (1.450) | 1.525 (1.450) |
| Vigorous-intensity pre-COVID-19 PA frequency | |  |  |
| Once a week or more |  | Reference | Reference |
| Less than once a week |  | **-0.773 (0.057) ***** | **-0.773 (0.057) ***** |
| No data |  | -1.713 (1.084) | -1.713 (1.084) |
| Health conditions^a^ |  |  |  |
| Diabetes |  | **-0.207 (0.071) **** | **-0.207 (0.071) **** |
| Cancer |  | 0.034 (0.085) | 0.034 (0.085) |
| Obesity |  | **-0.584 (0.059) ***** | **-0.584 (0.059) ***** |
| Heart disease |  | **0.181 (0.091)** * | **0.181 (0.091)** * |
| High blood pressure |  | **-0.185 (0.051) ***** | **-0.185 (0.051) **** |
| Asthma |  | 0.009 (0.066) | 0.009 (0.066) |
| Chronic lung disease |  | **-0.234 (0.111) *** | **-0.234 (0.111) *** |
| Kidney disease |  | **-0.381 (0.134) **** | **-0.381 (0.134) **** |
| Autoimmune disorder |  | -0.009 (0.090) | -0.009 (0.090) |
| Mental health condition |  | -0.031 (0.069) | -0.031 (0.069) |
| **State-level containment measures** | | | |
| Oxford COVID-19 Government Response Tracker Stringency Index | | | -0.000 (0.001) |

*Notes.* ^*^*p* < 0.05; ^**^*p* < 0.01; ^***^*p* < 0.001, PA = physical activity, ^a^ Reference category = does not have the particular health condition. Model 1 included only sociodemographic variables, Model 2 included sociodemographic variables and health-related behaviors and outcomes; Model 3 included sociodemographic variables, health-related behaviors and outcomes and containment measure stringency. The figures inside brackets are unstandardized regression coefficients, figures in brackets are standard errors. Weights provided by the UAS were used to adjust for the complex survey design, non-response rate, unequal selection probabilities and non-random attrition across waves. The Satterthwaite method was applied to the *t*-tests used for significance testing. All models were adjusted for survey wave and state of residence.

**Supplementary Table 2.** Unstandardized regression coefficients with standard error (in brackets) from Sensitivity Analysis 2: limiting analysis to those with complete data on pre-COVID-19 physical activity frequency (n=4903)

|  | Model 1 | Model 2 | Model 3 |
| --- | --- | --- | --- |
| **Sociodemographic characteristics** | | | |
| Age group (years) |  |  |  |
| 18-24 | Reference | Reference | Reference |
| 25-34 | -0.088 (0.077) | -0.035 (0.074) | -0.035 (0.074) |
| 35-44 | 0.040 (0.086) | 0.134 (0.082) | 0.134 (0.082) |
| 45-55 | **0.197 (0.091) *** | **0.356 (0.087) ***** | **0.354 (0.087) ***** |
| 55-69 | **0.340 (0.093) ***** | **0.544 (0.089) ***** | **0.543 (0.089) ***** |
| ≥70 | **0.583 (0.106) ***** | **0.822 (0.103) ***** | **0.820 (0.103) ***** |
| Sex |  |  |  |
| Male | Reference | Reference | Reference |
| Female | **-0.319 (0.053) ***** | **-0.188 (0.048) ***** | **-0.188 (0.048) ***** |
| Race |  |  |  |
| White | Reference | Reference | Reference |
| Non-White | **-0.358 (0.072) ***** | **-0.229 (0.064) ***** | **-0.229 (0.064) ***** |
| College degree |  |  |  |
| Yes | Reference | Reference | Reference |
| No | **-0.255 (0.050) ***** | -0.003 (0.046) | -0.002 (0.046) |
| Living with partner |  |  |  |
| Yes | Reference | Reference | Reference |
| No | **-0.133 (0.043) **** | -0.061 (0.040) | -0.060 (0.040) |
| Employment status |  |  |  |
| Employed | Reference | Reference | Reference |
| Retired | **0.322 (0.055) ***** | **0.302 (0.052) ***** | **0.303 (0.052) ***** |
| Unemployed | -0.033 (0.028) | **-**0.005 (0.028) | **-**0.005 (0.028) |
| Household income |  |  |  |
| ≤$15,000 | Reference | Reference | Reference |
| $15,001–$39,999 | **0.160 (0.039) ***** | **0.131 (0.038) ***** | **0.131 (0.038) ***** |
| $40,000–$99,999 | **0.108 (0.043) *** | 0.027 (0.042) | 0.027 (0.042) |
| ≥$100,000 | **0.242 (0.053) ***** | 0.083 (0.052) | 0.083 (0.052) |
| **Health-related behaviors and outcomes** | | | |
| Light-intensity pre-COVID-19 PA frequency | |  |  |
| More than once a week |  | Reference | Reference |
| Once a week or less |  | **-0.465 (0.056) ***** | **-0.465 (0.056) ***** |
| Moderate-intensity pre-COVID-19 PA frequency | |  |  |
| More than once a week |  | Reference | Reference |
| Once a week or less |  | **-0.930 (0.060) ***** | **-0.930 (0.060) ***** |
| Vigorous-intensity pre-COVID-19 PA frequency | |  |  |
| More than once a week |  | Reference | Reference |
| Once a week or less |  | **-0.840 (0.057) ***** | **-0.840 (0.057) ***** |
| Health conditions^a^ |  |  |  |
| Diabetes |  | -0.122 (0.077) | -0.122 (0.077) |
| Cancer |  | -0.003 (0.092) | -0.003 (0.092) |
| Obesity |  | **-0.458 (0.064) ***** | **-0.458 (0.064) ***** |
| Heart disease |  | **0.208 (0.100) *** | **0.209 (0.100) *** |
| High blood pressure |  | **-0.129 (0.057) *** | **-0.129 (0.057) *** |
| Asthma |  | 0.083 (0.073) | 0.083 (0.073) |
| Chronic lung disease |  | **-0.199 (0.121)** | **-0.199 (0.121)** |
| Kidney disease |  | **-0.300 (0.152) *** | **-0.301 (0.152) *** |
| Autoimmune disorder |  | -0.107 (0.099) | -0.107 (0.099) |
| Mental health condition |  | 0.007 (0.075) | 0.007 (0.075) |
| **State-level containment measures** | | | |
| Oxford COVID-19 Government Response Tracker Stringency Index | | | -0.001 (0.001) |

*Notes.* ^*^*p* < 0.05; ^**^*p* < 0.01; ^***^*p* < 0.001, PA = physical activity, ^a^ Reference category = does not have the particular health condition. Model 1 included only sociodemographic variables, Model 2 included sociodemographic variables and health-related behaviors and outcomes; Model 3 included sociodemographic variables, health-related behaviors and outcomes and containment measure stringency. The figures inside brackets are unstandardized regression coefficients, figures in brackets are standard errors. Weights provided by the UAS were used to adjust for the complex survey design, non-response rate, unequal selection probabilities and non-random attrition across waves. The Satterthwaite method was applied to the *t*-tests used for significance testing. All models were adjusted for survey wave and state of residence.
